# Mr. Moore, on the Vascularity of the Teeth

**Published:** 1803-10-01

**Authors:** Samuel Moor

**Affiliations:** No. 6, Palsgrave Place, Temple


### *To the Editors of the Medical and Physical Journal.*

GENTLEMEN,

I Take the liberty of communicating to you, a few observations on a late publication, entitled, "The Natural History of the human Teeth, by Mr. Joseph Fox.

In page 32, 33, and 34, the author labours to establish a new system of his own, which is the vascularity in the solid substance of the teeth.

Page 32, he says, "There is another set of vessels called absorbents, of the existence of which, in the structure of common bone, I believe there is no doubt, and on account of certain effects produced upon the teeth, we must conclude that they are not destitute of them."

This

This is not conclusive; the author knows that the teeth have not the same advantage in the animal economy that other bones have; their formation, growth, and manner of ossification, differ from all other bones, and therefore must be considered as different in their nature and action. They have neither a cartilaginous nor a membranous base to form on, but on a pulp peculiar to themselves; they are not cellular, but compact in their structure, and cannot be considered as other bones.

In the same page the author says, " In some cases we find the teeth undergo the ulcerative process, and a considerable quantity of the inner part is removed; a circumstance which could not happen, unless there were absorbents entering into the cavities of the teeth, and properly belonging to them."

This circumstance, in my opinion, is occasioned by a different process, and arises from distinct causes, though similar in their action and effect. One is, when the vessels and membrane, which are in the cavities of the roots, become inflamed, suppuration takes place, and the pus so formed cannot be removed from those parts, as they have no absorbents to take it up. Hence it becomes acrid, and destroys the internal texture of the tooth. The same process takes place, but in a greater degree, in young subjects, when the remaining pulp in the fang has not as yet finished the inner part of the tooth, and either from external violence or internal cause becomes disturbed; this process takes place, and a larger portion of pus is formed; and when such teeth are extracted, and a longitudinal section is made, the internal substance so decomposed, is found in the state of a moist black powder.

Further on, in the same page, the author advances as a conclusive argument in proof of the vascularity of the teeth, the following observations.

" Besides these instances, the effects of absorption in the tusks of elephants are often seen; sometimes in sawing these bodies, iron balls, spear heads, &c. are met with, which have been forced into them in attempting to kill these animals. These extraneous substances are always found loose, having a space in which they can be moved; this could never happen, unless there were some action going on, by which part of the bone could be removed; and there is no other mode in which it can be effected, but through the medium of the absorbent vessels."

This I consider as a feeble support of the author's system. But allowing for a moment the existence of absorbents, why why does not the arterial system renovate the absorbed portion? As this is not the case, it is evident that the teeth neither possess the one nor the other.

But the small vacuity which allows motion to the iron bodies, in the part they are found, is easily accounted for, without having recourse to absorption.

When the animal was wounded, the ball, by its velocity, as it entered the substance of the tusk, must destroy the cohesive power of that part it comes in contact with; the animal lives for many years afterwards, and ranges about; the air is admitted into the orifice, and moisture also; rust forms on those iron bodies, and thereby their circumference is diminished, and at the same time destroys a portion of the bony lamella; of course, that orifice becomes enlarged, and allows motion to those bodies. Had the animal lived still longer, a larger portion of the bony substance would have been lost, so as to permit those bodies to drop out.

The teeth being deprived of animal vitality in their substance, cannot constitutionally be acted upon; all the alteration the teeth undergo, is from a chemical or a mechanical process, and can only be considered as a defect, and not a disease.

They never assume any of the diseases of the other bones, and the following argument will favor this hypothesis. They have no power to exfoliate any fractured portion, nor renovate their wasted substance. The gout, rheumatism, venereal disease, or the inveterate scurvy, do not alter their structure, nor does any ichor discharge from the diseased part; they form no exostosis, nor are they affected by the spina ventosa; they are only liable to one peculiar defect, which is a caries, and this from a chemical or mechanical process, and not constitutional. They are entirely passive, and have no power of resistance; the same process of decay takes place on the human teeth, supplied by art, or those made of the hippopotamus; this is decisive that the caries is not a constitutional disease.

Page 34, the author says, "A large quantity of blood is distributed to the teeth; this may frequently be seen in performing some operations. In cutting off the crown of a tooth, in which the caries had not spread to the fang, for the purpose of engrafting a new tooth, I have several times seen a discharge of blood from the internal cavity."

But in my practice, I have never seen, nor will the author say he has, that any blood was discharged from the solid solid surrounding part of the fang, or body of the tooth, nor any sensation perceived, unless the instrument employed, came in contact with the vessels in the cavity. The lateral branches of those vessels are expanded on the internal membrane and there are lost, without entering the bony substance.

In the same page the author says, "Blood carries with it the basis of nutrition, and is sent to those parts only where renovation is necessary. For what other reason then but to impart some principle of nutrition, can so much blood flow into the teeth? If the teeth, after their first formation, received no supply from vessels, or did not require any nourishment, it would have been better if they had been destitute of an internal cavity, and of regular organization."

This will serve as an additional proof of the non vascularity of the teeth, as they never renovate any lost portion; their situation would not allow of that process, and the vessels would be useless; they must be considered in their substance as inorganized bodies.

As an additional proof, the author says, "It is always observed, that as persons advance in life, their teeth lose that whiteness which they possessed in the time of youth. This change in the appearance of the teeth seems to depend upon one which takes place in their cavities, by which the vessels entering them are gradually destroyed, and the supply of blood is proportionally diminished. In the teeth of persons advanced in years, the cavity is very frequently obliterated, in consequence of a deposit of bony matter, which entirely destroys the internal organization. When this happens, the teeth always lose their colour, and become very yellow."

But the alteration of colour which the teeth undergo, arises from a mechanical and chemical process. The friction on the bases of our grinders, occasioned by mastication, and by the friction of our lips, food, and by cleaning, diminishes by degrees the enamel on their surface and sides, and the bony part will appear through the enamel. The adult teeth, which during a long period of life have been acted upon by the juices of the mouth, heat of the body, by the various food, solid and liquid, undergo in addition to the mechanical operation, a chemical process, and by that means lose their primitive whiteness. And that teeth do undergo a discolouration in their substance, by a chemical process, may be seen by those who have accustomed accustomed themselves to smoking, and particularly chewing of tobacco.

Page 35. The proof that the author further adduces for the internal circulation, is, "When a tooth has been loosened by a blow, and has afterwards fastened in its socket, a great alteration in its colour is the consequence; it gradually loses its whiteness, and acquires a darker hue, because it receives no longer any blood."

But the reason for the discolouration in this case, proceeds from an extravasation from the ruptured vessels, the blood lodges within the cavity, and acts as a foil to the bony part of the tooth.

When the absorption takes place in children's teeth, and they are of course cut off from the main branches of the vessels which supply them, they cannot receive any influx of blood, and still retain their primitive whiteness; but this is on account of their short duration, and the simple food on which children live during that period; no chemical action could affect them materially in so short a time.

The teeth are not liable to any disease but one, which is peculiar to themselves, namely a caries. This never begins internally, except in two cases already mentioned; the decay of the teeth has its origin always externally, which by small degrees destroys the attractive, cohesive power of the component parts, and that process goes on, sometimes very slowly, and at others with great rapidity.

When a tooth becomes carious, we are not apprized by any internal sensitive symptoms, we perceive no pain, as no diseased vital action can go on, and no pain is perceived till the cavity is laid open, and the air, or any heterogeneous particles, are admitted to come in contact with the vessels within.

The author says farther on, that "the teeth suffer like other bones, as they are subject to that species of inflammation, called the ossific; by this means the fangs become increased in size, and exhibit the appearances of exostosis.

But this appearance is a deception, and arises not from an inflammation, but is natural, or a malformation, and that it is so, may be seen by several sound teeth of all classes, which I have in my possession perfect without any defect, and some of very young subjects, recently formed, and which were extracted by myself, for want of room in the circle of the jaw.

Besides, what the author calls an exostosis appearance, from the ossific inflammation, the same often appears, and of much larger extent, on the bodies of the grinders, bicuspides, pides, and I have also a small incisor by me which has the same on the concave part of the body; and surely the enamel of those teeth could not have those appearances from an ossific inflammation.

If the fangs were capable of an increase by the ossific inflammation, they would increase at times to an extraordinary size, and the socket would not be able to contain them; according to its original formation, the tooth would become loose. I have extracted a number of teeth with those appearances, and all of them perfectly firm in their socket.

What has been advanced in the above pages, against the vascularity, will plainly demonstrate that the teeth are not vascular in their solid substance, and of course cannot become constitutionally diseased.

*No. 6, Palsgrave Place, Temple,* I am, &c.

